# Generalized Softened Variable Angle Truss Model for RC Hollow Beams under Torsion

**DOI:** 10.3390/ma12132209

**Published:** 2019-07-09

**Authors:** Luís Bernardo

**Affiliations:** C-MADE - Centre of Materials and Building Technologies, Department of Civil Engineering and Architecture, University of Beira Interior, 6201-001 Covilhã, Portugal; lfb@ubi.pt; Fax: +351-275-329-969

**Keywords:** RC Hollow Beams, torsion, STA, GSVATM

## Abstract

In recent studies, a new softened truss model called Generalized Softened Variable Angle Truss Model (GSVATM) has been proposed to compute the full torsional response of reinforced concrete (RC) rectangular solid beams under pure torsion. In this article, the GSVATM is extended to cover RC hollow beams under torsion. The modification of the calculation procedure, in order to account for the specific behavior of RC hollow beams for low loading levels, as well as the final solution procedure, is presented. The theoretical predictions from the extended GSVATM are compared with experimental results of RC hollow beams under torsion found in the literature. Good agreement is observed between the experimental and theoretical results, for both high and low loading levels.

## 1. Introduction

One of the most comprehensive and used analytical toll to model the behavior of reinforced concrete (RC) beams under torsion is the Space Truss Analogy (STA). Since the first model proposed by Rausch in 1929 [1], which was only able to predict the torsional strength of RC beams, further versions have been developed. Once STA allows the user to have a good understanding of how a RC beam behaves under torsion in the cracked stage, it has been used and developed by several researchers especially since the second half of the last century. Moreover, it constitutes the base model for most of current codes of practice for the design of RC beams under torsion. 

Although the general design rules of current codes of practice include orientations to assess structural beams for both the service (low) and ultimate (high) loads, specific design rules for torsion are still mainly focused on the ultimate limit state. For this reason, refined versions of the STA have been proposed which are able to predict accurately the torsional response of RC beams for all the loading levels, including for service loads.

One of the most recent refined STA models is the Generalized Softened Variable Angle Truss Model (GSVATM). The GSVATM was proposed by Bernardo et al. in 2015 [2] for RC rectangular solid beams under torsion. This analytical model constitutes a generalization of the Variable Angle Truss Model (VATM) proposed by Hsu and Mo in 1985 [3,4], which aimed to unify RC beams with small and large cross sections as well as RC and prestressed concrete (PC) beams under torsion. Since VATM neglects the contribution of the concrete tensile strength, which constitutes an important property in the early loading stages, this model is only able to predict correctly the behavior of RC beams under torsion for the ultimate stage, namely the torsional strength. GSVATM corrects this drawback by incorporating adequate smeared constitutive relationships for the materials [5], including for the tensile concrete, and shows to be able to predict the full torsional response of RC solid beams. More recently, the GSVATM was extended to cover PC beams [6] and RC beams under torsion combined with external axial forces [7], and the work is still ongoing. In general, the theoretical predictions from the GSVATM are in very good agreement with the experimental data of RC and PC rectangular solid beams under torsion for all loading levels, namely from the beginning of loading until failure.

**Figure 1:**
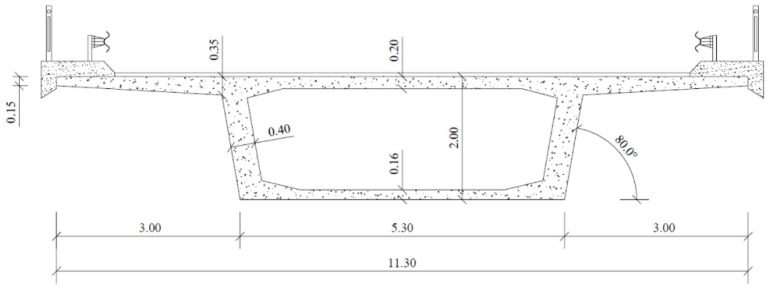
Example of a RC box (hollow) beam for a bridge deck (dimensions in meters).

Rectangular beams with hollow cross sections are used in many structural systems where large girders are used, such as long span bridges (Figure 1) and buildings with complex architecture. In such situations, very high bending and torsional moments are current. For high loads and long spans, beams with hollow cross sections are advantageous when compared with solid beams. For large cross sections under high bending moments or high torsional moments, the internal forces are mainly supported by the top and bottom concrete zone for bending or by the outer concrete shell for torsion. For these situations, the concrete at the center of the cross section is almost redundant. So hollow cross sections allow for a great reduction in weight and concrete consumption, with only a very small reduction of the bending and torsional strength. 

In reference [2], the GSVATM was only assessed for RC rectangular solid beams. The same happened in previous studies in which other refinements of the STA were proposed, such as the Softened Membrane Model for Torsion (SMMT) proposed by Jeng and Hsu in 2009 [8]. Some reasons can be pointed out to justify why RC hollow beams were excluded in such studies.

It has been known for a long time that the concrete core has negligible effect on the torsional strength of RC beams [9]. However, from previous studies it was also observed that the cracking twist, cracking torque, torsional stiffness and torsional ductility of hollow beams are lower than those of solid beams [8,10,11,12,13]. In fact, when used to predict the torsional behavior of RC hollow beams under torsion, both GSVATM and SMMT show in general worst predictions with highest dispersion of the results, mainly for the transition between the uncracked and cracked stage. This shows that such models need to be refined for RC hollow beams under torsion.

To assess any analytical model which aims to predict the behavior of RC beams under torsion, sufficient experimental results are needed to be compared with the theoretical predictions. However, the number of experimental results for RC hollow beams under torsion is limited when compared with the same ones related with RC solid beams. Hollow beams are more difficult to build and are more expensive. This explains why the majority of previous studies on the torsional behavior of RC beams that can be found in literature deal with RC solid beams.

From the aforementioned, it can be stated that specific studies with RC hollow beams must be performed when the objective is to assess analytical models which aim to predict the full behavior of such structural members under torsion, such as the GSVATM. Recently, additional experimental results related with RC hollow beams under torsion have been reported in literature [14] and which can help for this objective.

As previously referred, current codes of practice still incorporate specific and detailed rules for the design of RC cross sections under torsion mainly for the ultimate limit state. Much of the previously referred differences between RC hollow and solid beams are not yet accounted for. As a result, engineers still need to rely on personal judgement to properly design RC hollow beams under torsion.

From the above, it is clear that the theoretical modelling of RC hollow beams under torsion still needs further research.

In this article, the GSVATM is extended to cover RC hollow beams under torsion. The changes in the calculation procedure of the GSVATM, as well as the used solution procedure to compute the full response of such beams under torsion, is presented. The theoretical predictions from the GSVATM are also compared with experimental results of RC hollow beams under torsion found in the literature.

## 2. Brief Description of the GSVATM for RC Solid Beams

**Figure 2:**
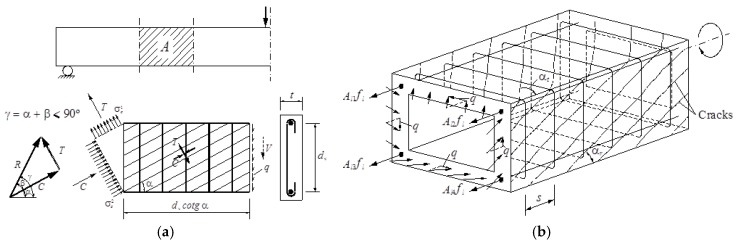
Beam elements modelled using the truss analogy: (**a**) thin beam, (**b**) box beam.

As previously referred, the GSVATM was initially proposed for RC solid beams under torsion. Full details about the GSVATM, assumptions, derivation of the equations and definition of the calculation and solution procedure can be found in reference [2]. Equations (1) to (5) govern the behavior of a RC thin beam element under a shear flow q (induced by a shear force *V*) which is modeled with a plain truss analogy (see beam element *A* in Figure 2a). To model an equivalent RC box beam element under torsion $M_{T}$ with the space truss analogy (Figure 2b), the walls are considered to be the union of four thin beam elements. From this assumption, Equations (6) to (11) are derived. The GSVATM incorporates a diagonal concrete strut with a compressive force *C*, with an angle α to the longitudinal axis, and a concrete tie with a tensile force *T* in the perpendicular direction to the concrete strut. The concrete strut and tie aim to simulate the resultant forces (*C* and *T*, respectively) due to the principal compressive and tensile stress fields in concrete ($\sigma_{2}^{c}$ and $\sigma_{1}^{c}$, respectively).

**Figure 3:**
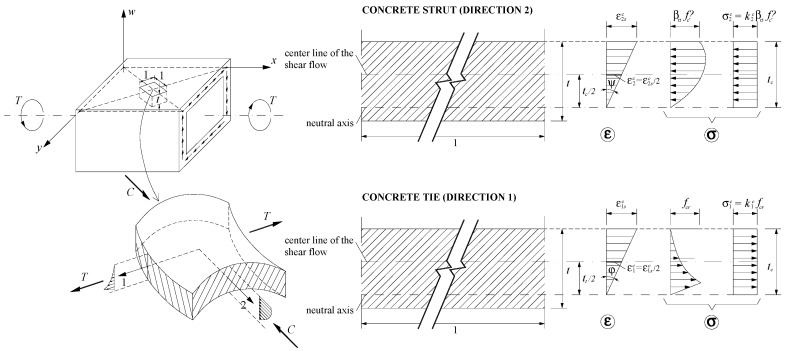
Strain gradient due the bending of the walls.

For the box beam, the torsional moment $M_{T}$ is related to the shear flow q by using the first equation from Bredt´s thin tube theory. The center line of the shear flow q is assumed to coincide with the center line of the walls with effective thickness $t_{c}$. Three equilibrium equations (Equations (6) to (8)) and three compatibility equations (Equations (9) to (11)) are derived for the equivalent box beam. If γ>90, Equation (7) is multiplied by (−1). Equation (12) is an invariant equation to relate the strains. The derivation of the equations accounts for the strain gradient in the walls due to bending (Figure 3).

The meaning of the parameters in Equations (1) to (12) are: *R* is the resultant force with angles *β* to the force *C* and γ to the longitudinal axis, *d_v_* is the distance between centers of the longitudinal bars, $t_{c}$ is the width of the cross section for the thin beam element and also the effective thickness of the concrete strut and tie in the walls of the box beam, $\varepsilon_{st}$ and $\varepsilon_{sl}$ are the strain in the transverse and longitudinal reinforcement, respectively, *θ* is the twist (torsional deformation), $\varepsilon_{2s}^{c}$ is the maximum compressive strain in the outer surface of the concrete strut, $\varepsilon_{1}^{c}$ and $\varepsilon_{2}^{c}$ are the average strains in the concrete strut and tie, respectively, *A*_0_ is the area limited by the center line of the shear flow q (*A*_0_ = (x − $t_{c}$) (y − $t_{c}$)), with *x* and *y* the minor and major outer dimension of the cross section), $p_{0}$ is the perimeter of the center line of the shear flow ($p_{0}$ = 2(x − $t_{c}$) + (y − $t_{c}$)), $A_{sl}$ is the total area of longitudinal steel, $A_{st}$ is the area of one bar of the transverse steel, *s* is the longitudinal spacing of the transverse reinforcement, $f_{sl}$ and $f_{st}$ are the stresses in the longitudinal and transverse reinforcement, respectively.

(1) $$R=\sqrt{C^{2}+T^{2}}$$

(2) β=arctan(T/C)

(3) γ=α+β

(4) $$C=\sigma_{2}^{c}t_{c}d_{v\;}\cos\alpha$$

(5) $$T=\sigma_{1}^{c}t_{c}d_{v\;}\sin\alpha$$

(6) $$M_{T}=\frac{2A_{0}Rsin\;\gamma }{d_{v\;}}$$

(7) $$t_{c}=\frac{A_{sl}f_{sl}}{\sigma_{2}^{c}p_{0}}\frac{\cos\beta }{\cos\alpha \cos\gamma }\;\mathrm{for}\;\gamma =\alpha +\beta \leq 90^{o}$$

(8) $$\alpha =\arctan\left[\frac{\sqrt{F^{2}\;{\left(\tan\beta \right)}^{2}+F\;{\left(\tan\beta \right)}^{4}+F+{\left(\tan\beta \right)}^{2}}}{F\;{\left(\tan\beta \right)}^{2}+1}\right]\;\mathrm{with}\;F=\frac{A_{st}f_{st}p_{0}}{A_{sl}f_{sl\;}s}$$

(9) $$\varepsilon_{st}=\left[\frac{A_{0}^{2}\sigma_{2}^{c}\sin\gamma }{p_{0}M_{T}\cos\beta \;\tan\alpha \sin\alpha }-\frac{1}{2}\right]\;\varepsilon_{2s}^{c}$$

(10) $$\varepsilon_{sl}=\left[\frac{A_{0}^{2}\sigma_{2}^{c}\sin\gamma }{p_{0}M_{T}\cos\beta \;\cot\alpha \sin\alpha }-\frac{1}{2}\right]\;\varepsilon_{2s}^{c}$$

(11) $$\theta =\frac{\varepsilon_{2s}^{c}}{2t_{c\;}\sin\alpha \cos\alpha }$$

(12) $$\varepsilon_{1s}^{c}=2\varepsilon_{1}^{c}=2\varepsilon_{sl}+2\varepsilon_{st}+2\varepsilon_{2s}^{c}$$

The behavior of the compressive concrete strut, tensile concrete tie and tensile longitudinal and transverse steel bars is modeled by using smeared stress (σ) – strain (ε) relationships. For RC beams under torsion, some suitable σ−ε relationships were found in a previous study [5]. For the compressive concrete, the softened σ−ε relationship proposed by Belarbi and Hsu in 1995 [15] (Equations (13) and (14) with softening factor $\beta_{*}$ = $\beta_{\sigma }$ = $\beta_{\varepsilon }$ for both the peak stress and corresponding strain proposed by Zhang and Hsu in 1998 [16] (Equations (15) to (18)) are used. For the tensile concrete, the stiffened σ−ε relationship proposed by Belarbi and Hsu in 1994 [17] and modified by Jeng and Hsu in 2009 [8] and Bernardo et al. in 2013 [12] (Equations (19) to (23)) is used. For the steel bars in tension, the stiffened σ−ε relationship proposed by Belarbi and Hsu in 1994 [17] (Equations (30) to (32)) is used. The meaning of the parameters are: $f_{c}^{'}$ is the average uniaxial concrete compressive strength, $\varepsilon_{0}$ is the strain corresponding to $f_{c}^{'}$, $\rho_{l}$ is the longitudinal reinforcement ratio ($\rho_{l}$ = $A_{sl}$/$A_{c}$, with $A_{c}$ = *xy*), $\rho_{t}$ is the transverse reinforcement ratio ($\rho_{t}$ = $A_{sl}$*u*/$A_{c}$*s*, with *u* = 2*x* + 2*y*), $f_{ly}$ and $f_{ty}$ are the yielding stress for the longitudinal and transverse reinforcement, respectively, $E_{c}$ is the Young´s Modulus for concrete, $f_{cr}$ is the concrete cracking stress and $\varepsilon_{cr}$ is the strain corresponding to $f_{cr}$, $f_{s}$ and $\varepsilon_{s}$ are the stress and strain in the steel bars, respectively, $E_{s}$ is the Young´s Modulus for steel, $f_{y}$ is the yielding stress of steel bars and ρ is the reinforcement ratio.

(13) $$\sigma_{2}^{c}=\beta_{\sigma }f_{c}^{'}\left[2\left(\frac{\varepsilon_{2}^{c}}{\beta_{\varepsilon }\varepsilon_{0}}\right)-{\left(\frac{\varepsilon_{2}^{c}}{\beta_{\varepsilon }\varepsilon_{0}}\right)}^{2}\right]\;\mathrm{if}\;\varepsilon_{2}^{c}\leq \beta_{\varepsilon }\varepsilon_{0}$$

(14) $$\sigma_{2}^{c}=\beta_{\sigma }f_{c}^{'}\left[1-{\left(\frac{\varepsilon_{2}^{c}-\beta_{\varepsilon }\varepsilon_{0}}{2\varepsilon_{0}-\beta_{\varepsilon }\varepsilon_{0}}\right)}^{2}\right]\;\mathrm{if}\;\varepsilon_{2}^{c}>\beta_{\varepsilon }\varepsilon_{0}$$

(15) $$\beta_{*}=\beta_{\sigma }=\beta_{\varepsilon }=\frac{R(f_{c}^{'})}{\sqrt{1+\frac{400\varepsilon_{1}^{c}}{\eta^{\prime }}}}$$

(16) $$\eta =\frac{\rho_{lf_{ly}}}{\rho_{tf_{ty}}}$$

(17) $$\{\begin{array}{c} \eta \leq 1\to \eta^{\prime }=\eta \\ \eta >1\to \eta^{\prime }=\frac{1}{\eta } \end{array}$$

(18) $$R(f_{c}^{'})=\frac{5.8}{\sqrt{f_{c}^{'}\left(\mathrm{MPa}\right)}}\leq 0.9$$

(19) $$\sigma_{1}^{c}=E_{c}\varepsilon_{1}^{c}\;\mathrm{if}\;\varepsilon_{1}^{c}\leq \varepsilon_{cr}$$

(20) $$\sigma_{1}^{c}=f_{cr}{\left(\frac{\varepsilon_{cr}}{\varepsilon_{2}^{c}}\right)}^{0.4}\;\mathrm{if}\;\varepsilon_{1}^{c}>\varepsilon_{cr}$$

(21) $$E_{c}=3875K\sqrt{f_{c}^{'}\left(\mathrm{MPa}\right)}$$

(22) $$\varepsilon_{cr}=0.00008K$$

*K* = 1.45 (solid beams) (23)

(24) $$\sigma_{2}^{c}=k_{2}^{c}\beta_{\sigma }f_{c}^{'}$$

(25) $$k_{2}^{c}=\frac{\varepsilon_{2s}^{c}}{\beta_{\varepsilon }\varepsilon_{0}}-\frac{\left(\varepsilon_{2s}^{c}\right){\;}^{2}}{3\left(\beta_{\varepsilon }\varepsilon_{0}\right){\;}^{2}}\;\mathrm{if}\;\varepsilon_{2s}^{c}\leq \beta_{\varepsilon }\varepsilon_{0}$$

(26) $$k_{2}^{c}=1-\frac{\beta_{\varepsilon }\varepsilon_{0}}{3\varepsilon_{2s}^{c}}-\frac{{\left(\varepsilon_{2s}^{c}-\beta_{\varepsilon }\varepsilon_{0}\right)}^{3}}{3\varepsilon_{2s}^{c}{\left(2\varepsilon_{0}-\beta_{\varepsilon }\varepsilon_{0}\right)}^{2}}\;\mathrm{if}\;\varepsilon_{2s}^{c}>\beta_{\varepsilon }\varepsilon_{0}$$

(27) $$\sigma_{1}^{c}=k_{1}^{c}f_{cr}$$

(28) $$k_{1}^{c}=\frac{\varepsilon_{1s}^{c}}{2\varepsilon_{cr}}\;\mathrm{if}\;\varepsilon_{1s}^{c}\leq \varepsilon_{cr}$$

(29) $$k_{1}^{c}=\frac{\varepsilon_{cr}}{2\varepsilon_{1s}^{c}}+\frac{\left(\varepsilon_{cr}\right){}^{0.4}}{0.6\varepsilon_{1s}^{c}}\left[\left(\varepsilon_{1s}^{c}\right){}^{0.6}-\left(\varepsilon_{cr}\right){}^{0.6}\right]\;\mathrm{if}\;\varepsilon_{1s}^{c}>\varepsilon_{cr}$$

(30) $$f_{s}=\frac{0.975E_{s}\varepsilon_{s}}{{\left[1+{\left(\frac{1.1E_{s}\varepsilon_{s}}{f_{y}}\right)}^{m}\right]}^{\frac{1}{m}}}+0.025E_{s}\varepsilon_{s}$$

(31) $$m=\frac{1}{9B-0.2}\leq 25$$

(32) $$B=\frac{1}{\rho }{\left(\frac{f_{cr}}{f_{y}}\right)}^{1.5}$$

The stresses $\sigma_{2}^{c}$ and $\sigma_{1}^{c}$ are the average stress of non-uniform stress diagrams in the concrete strut (Equation (24)) and concrete tie (Equation (27)), respectively, due to the gradient of the strains in the walls (Figure 3). Parameters $k_{2}^{c}$ (Equations (25) and (26)) and $k_{1}^{c}$ (Equations (28) and (29)) are computed from integration of Equations (13)–(14) and (19)–(20), respectively.

**Figure 4:**
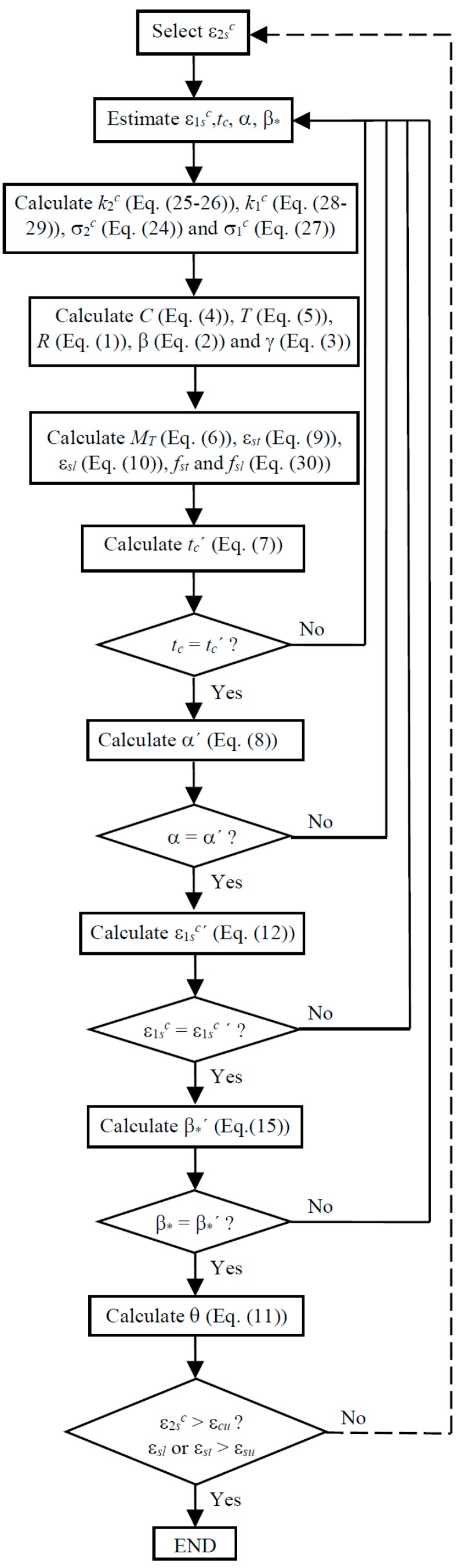
GSVATM flowchart for RC solid beams.

The GSVATM is used to compute the theoretical $M_{T}$−*θ* curve of RC solid beams. For this, a trial-and-error technique is used to establish a nonlinear solution procedure. The first input value, among other ones, is the strain at the outer fiber of the concrete strut $\varepsilon_{2s}^{c}$ = 2$\varepsilon_{2}^{c}$ (see Figure 3). This parameter is incremented from the previous one for each new cycle. At the end of each cycle a solution point for the theoretical $M_{T}$−*θ* curve is calculated. Figure 4 shows the flowchart for the iterative calculation algorithm, which was implemented in computer by using programming language Delphi [2].

The calculation procedure of the GSVATM ends when the conventional failure of the beam is reached. For this, conventional ultimate (failure) strains need to be assumed for both concrete in compression ($\varepsilon_{cu}$) and steel in tension ($\varepsilon_{su}$)).

## 3. Calculation Procedure for RC Hollow Beams under Torsion

This section aims to present the modified calculation procedure to model RC hollow beams under torsion by using the GSVATM.

### 3.1. Introduction

Jeng in 2015 [14] proposed a modified calculation procedure to refine the SMMT, which was previously proposed by Jeng and Hsu in 2009 [8] for RC solid beams under torsion. The SMMT constitutes an extension of the Softened Membrane Model (SMM) proposed by Hsu and Zhu in 2002 [18]. Jeng carried out a series of additional experiments on RC hollow beams under torsion (5 hollow beams with thin walls and 4 hollow beams with thick walls), which enabled to increase the number of available experimental data. Moreover, such experiments were especially designed as highly controlled to accurately record the behavioral stage of the beams prior to cracking, as well as the cracking torque and the corresponding twist. With these additional experimental results, it was possible for Jeng to specifically calibrate some parameters of the constitutive relationships for concrete in tension and compression used in the original model, in order to consider the specific behavior under torsion of RC hollow beams [14]. To calibrate the referred parameters, Jeng found the need to establish a new classification for hollow beams, namely "thin wall" hollow beams and "thick wall" hollow beams. In addition, Jeng limited the thickness of the shear flow in the walls of the hollow cross section. This allowed the author to extend the SMMT for RC rectangular hollow beams. Good results were observed when the predictions of the model were compared with the experimental data. 

In this study, a similar calculation procedure as the one previously referred is implemented to modify the GSVATM, with the aim to unify the model for both RC rectangular solid and hollow beams. This option can be justified because both SMMT and GSVATM are STM based models and it is expected that the changes in SMMT to cover RC hollow beams are also valid for the GSVATM. The changes in GSVATM are assessed in Section 5 by using the experimental results of RC rectangular hollow beams tested under torsion until failure that were found in the literature, including the additional ones tested by Jeng and reported in 2015 [14].

### 3.2. Thickness of the Shear Flow

When the STA is used to model a RC beam under torsion, the effective thickness $t_{c}$ of the walls of the equivalent box beam is assumed to be equal to the thickness of the shear flow q (Figure 2b). This is also valid for both SMMT and GSVATM models.

Before cracking, the thickness $t_{c}$ is constant. Right after cracking, this thickness highly decreases and remains approximately constant until failure [19,20]. The small change on the thickness $t_{c}$ after cracking explains why the ultimate torque of RC hollow beams is similar to RC solid beams. In fact, when the torsional moment reaches its maximum value, thickness $t_{c}$ is usually smaller than the real wall thickness t of RC hollow beams. When using GSVATM or SMMT to model RC hollow beams, it is observed that, in the stage before cracking, the calculated thickness $t_{c}$ is often higher than the real wall thickness t [14,21]. This observation partially explains the differences between the theoretical predictions and the experimental results for such beams.

According to Jeng in 2015 [14], for a RC hollow beam with a real wall thickness t ≤ $t_{c,solid}$, with $t_{c,solid}$ being the value for thickness $t_{c}$ computed for each level torque with GSVATM (Equation (7)) for the equivalent RC solid beam (beam with same outer dimensions, same reinforcement and same materials), it must be imposed in the calculation procedure that the thickness of the shear flow zone occupies the entire real thickness of the wall, i.e., $t_{c}$ = t With this criterion the thickness $t_{c}$ is thus limited by the real wall thickness t of the RC hollow beam. 

In addition, and according to Jeng in 2015 [14], it is also necessary to calibrate the constitutive relationships for concrete, as presented in the next subsection. For this, it is necessary to establish a new classification for the RC hollow beam according to whether the thickness of the wall corresponds to a "thin wall" or a "thick wall". This distinction makes it possible, according to Jeng, to better carry out the necessary corrections in the constitutive relationships for concrete, either in tension or in compression. For this, the RC hollow beam is firstly calculated as an equivalent RC solid beam by using the original unmodified model until the cracking torque $M_{Tcr,solid}$ is reached. By knowing the corresponding value for the effective thickness $t_{c,cr,solid}$, the following classification is applied (being t the real thickness of the wall):

- if t ≤ 0.91$t_{c,cr,solid}$ the RC hollow beam has a “thin wall”;
- if t ≤ 0.91$t_{c,cr,solid}$ the RC hollow beam has a “thick wall”.

### 3.3. Constitutive Relationships for Concrete

By using both the GSVATM and the SMMT with the constitutive relationships presented in Section 2 it is observed that the obtained values for the cracking torque $M_{Tcr}$ are highly overestimated for RC hollow beams [8,21]. To solve this drawback, Jeng in 2015 [14] proposed new correction parameters for the constitutive relationship equations for both concrete in compression and tension, namely parameters *η*, *μ* and *λ*. The values of these new parameters depend on the type of cross section (solid beam, thin-walled or thick-walled hollow beam) and can be calculated from the following Equations [14]:

(33) $$\sigma_{2}^{c}=\eta k_{2}^{c}\beta_{\sigma }f_{c}^{'}$$

(34) $$\sigma_{1}^{c}=\eta k_{1}^{c}f_{cr}$$

(35) $$\varepsilon_{cr}=0.00008\mu$$

(36) $$E_{c}=3875\lambda \sqrt{f_{c}^{'}\left(\mathrm{MPa}\right)}$$

Equations (33)–(36) substitute Equations (21), (22), (24) and (27), respectively.

For RC thin-walled hollow beams, the additional experimental tests performed and reported by Jeng [14] with 5 RC hollow beams with thin walls allowed him to calibrate rigorously both parameters *μ* and *λ* in order to approximate the experimental and theoretical (from SMMT) cracking torques $M_{Tcr}$. The final proposed values were the following ones:

(37) μ=λ=0.93 (RC thin−walled hollow beams)

From the experimental results of reference RC hollow beams found in the literature and previously classified as thin-walled hollow beams, Jeng performed a parametric analysis relating the ratio of the experimental to the theoretical (from SMMT) cracking torques with the compressive concrete strength. From a linear regression analysis, the following equation for correction parameter *η* was proposed [14]:

(38) $$\eta =0.033\sqrt{f_{c}^{'}\left(\mathrm{MPa}\right)}+0.73\;\left(\mathrm{RC}\;\mathrm{thin}-\mathrm{walled}\;\mathrm{hollow}\;\mathrm{beams}\right)$$

For RC thick-walled hollow beams, the additional experimental tests reported by Jeng [14] with 4 RC hollow beams with thick walls and also additional numerical results allowed him to calibrate the correction parameters. The following equations were proposed [14]:

(39) $$\mu =\lambda =1.20\;\left(\mathrm{RC}\;\mathrm{thick}-\mathrm{walled}\;\mathrm{hollow}\;\mathrm{beams}\;\mathrm{and}\;f_{c}^{'}\leq 47.85\;\mathrm{MPa}\right)$$

(40) $$\eta =0.0938\sqrt{f_{c}^{'}\left(\mathrm{MPa}\right)}+0.43\;(\mathrm{RC}\;\mathrm{thick}-\mathrm{walled}\;\mathrm{hollow}\;\mathrm{beams}\;\mathrm{and}\;f_{c}^{'}\leq 47.85\;\mathrm{MPa})$$

(41) $$\mu =\lambda =1.129\;(\mathrm{RC}\;\mathrm{thick}-\mathrm{walled}\;\mathrm{hollow}\;\mathrm{beams}\;\mathrm{and}\;f_{c}^{'}>47.85\;\mathrm{MPa})$$

(42) $$\eta =\frac{8.45}{\sqrt{f_{c}^{'}\left(\mathrm{MPa}\right)}}+0.017\;(\mathrm{RC}\;\mathrm{thick}-\mathrm{walled}\;\mathrm{hollow}\;\mathrm{beams}\;\mathrm{and}\;f_{c}^{'}>47.85\;\mathrm{Mpa})$$

For RC solid beams, since Equations (22) and (23) remain valid, the previous correction parameters can be assumed to be:

*μ* = *λ* = 1.45 and *η* = 1 (RC solid beams) (43)

The previous proposed correction parameters from Jeng in 2015 [14] were also adopted in this study to extend the GSVATM for RC hollow beams under torsion.

### 3.4. New Calculation Procedure for the GSVATM

**Figure 5:**
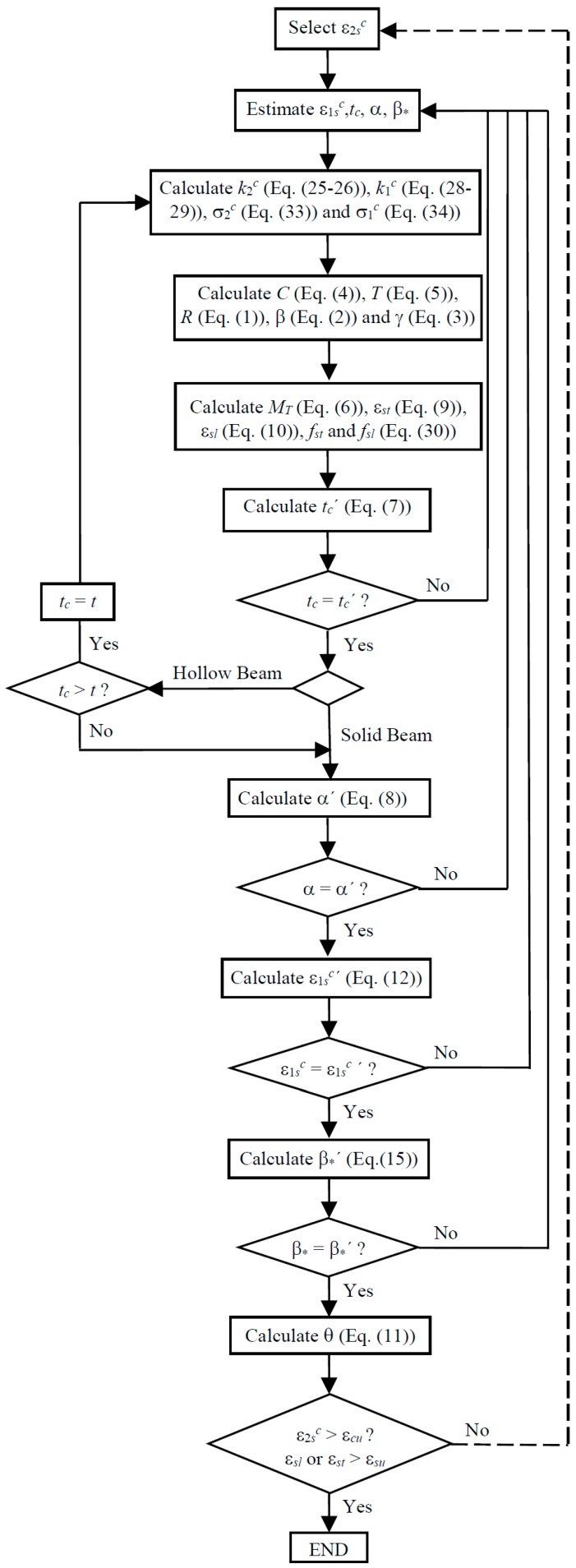
New flowchart for GSVATM for RC solid and hollow beams.

Based on the previous subsections, changes have been incorporated to the original calculation procedure of GSVATM in order to unify the analytical model for both RC solid and hollow beams under torsion. The new flowchart for the iterative calculation algorithm for GSVATM is shown in Figure 5, which is valid for both RC solid and hollow beams. Changes incorporate the correction parameters in the equations for the constitutive relationships of concrete (Equations (24) and (27) are substituted by Equations (33) and (34), respectively). In the new calculation algorithm, a RC hollow beam is initially calculated as an equivalent RC solid beam. For each value calculated for the thickness $t_{c}$ it is verified if $t_{c}>t$, i.e., if the thickness of the concrete strut (or the thickness of the shear flow) is higher than the real thickness of the concrete wall. If so, it is imposed that $t_{c}=t$ and the calculation procedure proceeds. If not, the calculation procedure proceeds with the equivalent RC solid beam.

## 4. Reference Beams

To assess the changes in the GSAVTM, as presented in the previous section, a comparative analysis between the predictions of the extended GSVATM and experimental data is performed in Section 5. For this, experimental results of reference RC hollow beams tested in pure torsion were compiled from the scientific literature [21]. From the reference beams found in the literature, beams with atypical failure under torsion (for instance, beams with insufficient torsional reinforcement or beams with atypical reinforcement detailing) were disregarded. Thirty reference beams were found for which all the data required to compute the $M_{T}$−θ curve with GSVATM were given, including the recent ones tested and reported by Jeng [14].

**Figure 6:**
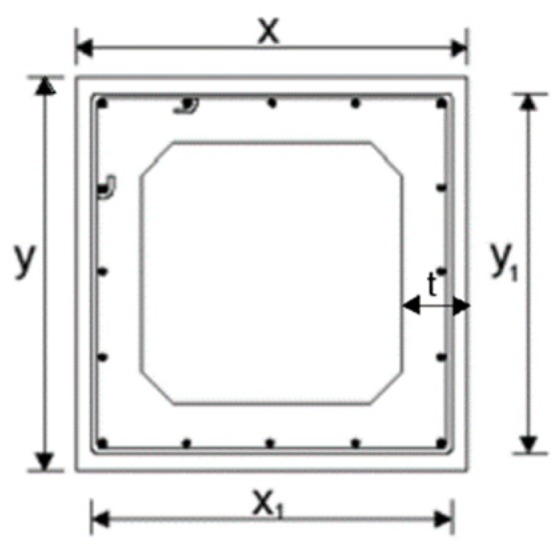
Geometrical parameters of the cross section.

Table 1 summarizes the properties of the reference beams. The geometrical parameters of the cross-section are defined in Figure 6.

In Table 1 the meaning of the parameters are: *t* is the thickness of the walls (see Figure 6), *x* and *y* are the external width and height, respectively, of the cross section (see Figure 6), *x*_1_ and *y*_1_ are the width and height, respectively, of the center line of the closed stirrup (see Figure 6), *A_sl_* is the total area of the longitudinal reinforcement, $A_{st}$ is the area of one bar of the transverse steel reinforcement, s is the longitudinal spacing of the transverse reinforcement, $\rho_{l}$ and $\rho_{t}$ are, respectively, the longitudinal and transverse reinforcement ratio ($\rho_{l}$ = $A_{sl}$/xy) and $\rho_{t}$ = 2$A_{sl}$(*x*_1_ + *y*_1_)/xys, $f_{ly}$ and $f_{ty}$ are, respectively, the yielding stresses of the longitudinal and transverse steel reinforcement and $f_{c}$ is the concrete compressive strength. Other necessary properties of the materials, such as the Young´s Modulus, conventional failure strains and concrete tensile strength were defined or computed according to Eurocode 2 [25].

## 5. Comparative Analyzes

In this section, comparative analyzes are performed between the theoretical results obtained from the extended GSVATM, as presented in Section 2 and Section 3, with the experimental results of the reference RC hollow beams presented in Section 4. Such comparative analyzes include the $M_{T}$−*θ* curves and some key points, namely the ones corresponding to the cracking and maximum torque.

Figure 7, Figure 8, Figure 9, Figure 10, Figure 11, Figure 12, Figure 13, Figure 14, Figure 15, Figure 16, Figure 17, Figure 18, Figure 19, Figure 20, Figure 21, Figure 22, Figure 23, Figure 24, Figure 25, Figure 26, Figure 27, Figure 28, Figure 29, Figure 30, Figure 31, Figure 32, Figure 33, Figure 34, Figure 35 and Figure 36 present, for each reference beams, the experimental and theoretical $M_{T}$−*θ* curves. With few exceptions, it can be stated that Figure 7, Figure 8, Figure 9, Figure 10, Figure 11, Figure 12, Figure 13, Figure 14, Figure 15, Figure 16, Figure 17, Figure 18, Figure 19, Figure 20, Figure 21, Figure 22, Figure 23, Figure 24, Figure 25, Figure 26, Figure 27, Figure 28, Figure 29, Figure 30, Figure 31, Figure 32, Figure 33, Figure 34, Figure 35 and Figure 36 show good agreement between the experimental and theoretical $M_{T}$−*θ* curves. The theoretical curves capture well the behavior of the reference beams for all loading levels. Both cracking torque and ultimate (maximum) torque show also good agreement and with small variability. 

**Figure 9:**
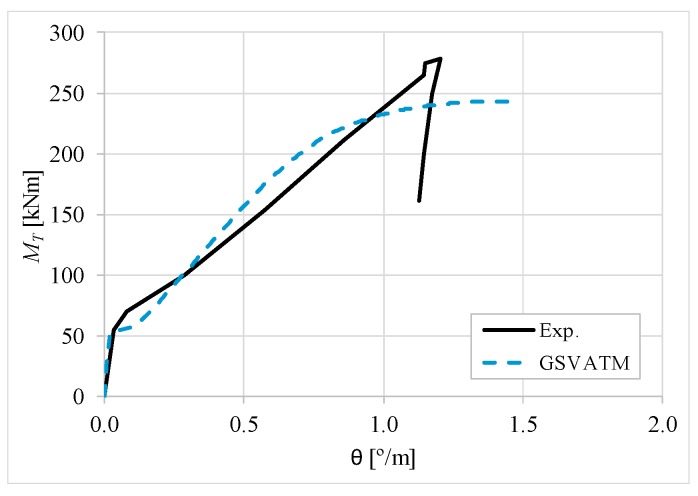
$M_{T}$−θ curves for Beam B065b.

**Figure 12:**
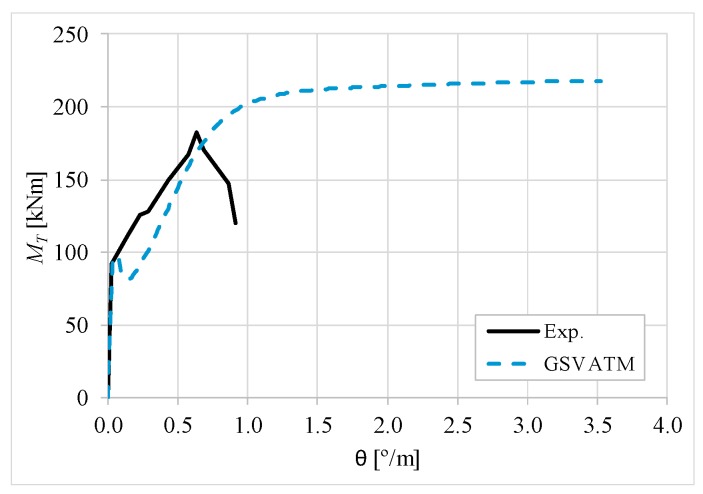
$M_{T}$−θ curves for Beam C065a.

**Figure 21:**
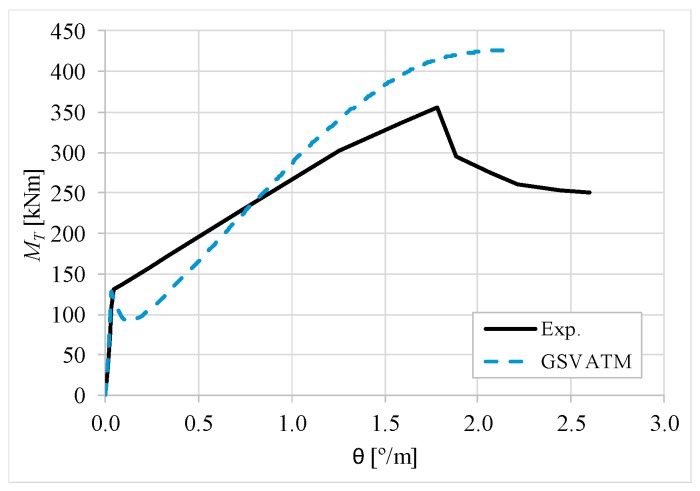
$M_{T}$−θ curves for Beam B3.

**Figure 25:**
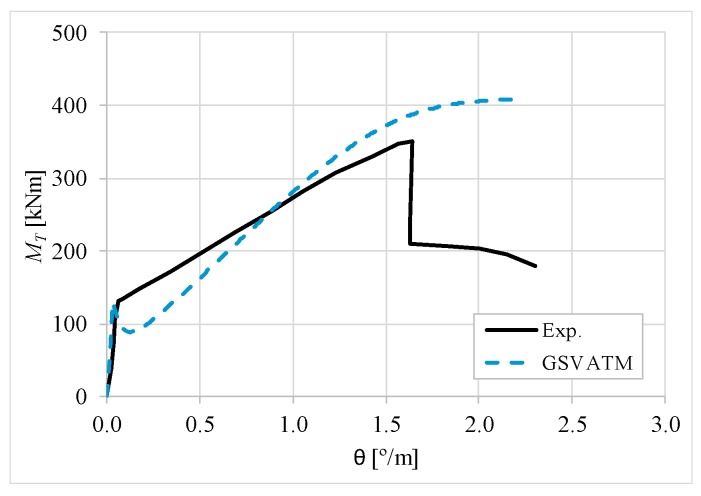
$M_{T}$−θ curves for Beam C3.

As far as the torsional strength is concerned, it should be referred that, according to the respective authors, Beams C065a (Figure 12), B3 (Figure 21) and C3 (Figure 25) suffered a somewhat premature failure [14,22]. This is clearly shown in the corresponding experimental $M_{T}$−*θ* curves. On the other hand, Beam B065b (Figure 9) suffered a sudden and unexpected failure which led to the somewhat weird final part of the corresponding experimental $M_{T}$−*θ* curve. These referred problems explain the differences observed between the theoretical and experimental torsional strength for such beams.

Figure 7, Figure 8, Figure 9, Figure 10, Figure 11, Figure 12, Figure 13, Figure 14, Figure 15, Figure 16, Figure 17, Figure 18, Figure 19, Figure 20, Figure 21, Figure 22, Figure 23, Figure 24, Figure 25, Figure 26, Figure 27, Figure 28, Figure 29, Figure 30, Figure 31, Figure 32, Figure 33, Figure 34, Figure 35 and Figure 36 also show another peculiar behavior. For most of the beams, the theoretical $M_{T}$−*θ* curves show a drop of the torsional moment right after the cracking torque. This behavior reflects the drop right after the peak stress in the smeared σ−ε relationship for tensile concrete (Equations (19) and (20)). This observation was also stated and discussed in detail in previous studies from the author [2], and also from other ones which use a different base model but with a similar constitutive relationship for tensile concrete [8].

Table 2 presents a comparative analysis between the numerical values corresponding to two key points of the $M_{T}$−*θ* curves, the cracking and ultimate points. Table 2 presents the theoretical and experimental values for the cracking torque ($M_{Tcr,th}$ and $M_{Tcr,exp}$), the twist corresponding to the cracking torque ($\theta_{cr,th}$ and $\theta_{cr,exp}$), the ultimate (maximum) torque ($M_{Tu,th}$ and $M_{Tu,exp}$) and the twist corresponding to the ultimate torque ($\theta_{u,th}$ and $\theta_{u,exp}$). For the two last parameters, reference beams C065a, B3 and C3 were not included for the comparative analysis for the reasons stated before. Table 2 also presents the ratio between the experimental to the theoretical values ($M_{Tcr,exp}$/$M_{Tcr,th}$, $\theta_{cr,exp}$/$\theta_{cr,th}$, $M_{Tu,exp}$/$M_{Tu,th}$ and $\theta_{u,exp}$/$\theta_{u,th}$), as well as the corresponding average value ($\bar{x}$), standard deviation (s) and correlation coefficient (*cv*).

The results from Table 2 confirm that GSVATM provides very good predictions with low variability for both the cracking torque ($\bar{x}$ = 1.033 and *cv* = 9.46%) and the ultimate torque ($\bar{x}$ = 1.033 and *cv* = 7.52%). For the corresponding twists, the predictions are less good and with higher variability, mainly for the twist corresponding to the cracking torque ($\bar{x}$ = 1.601 and *cv* = 44.42%). The values for the experimental twist are very small in the non-cracked stage. The accuracy limitation of the rotational gauges used in the experiments can probably explain the observed results for the cracking twist. For the twist corresponding to the ultimate torque, despite the average value is good ($\bar{x}$ = 0.954), the variability is somewhat high (*cv* = 27.03%). It is known that the deformations in the ultimate stage are more difficult to capture correctly with analytical models due to the complexity of modelling the effect of the damage in materials. Such observation was also observed in previous studies [2,8,14]. Since the cracking and ultimate twists are not very important for the design, the worst results related with these parameters can be considered less important.

In general, the results previously presented agree with the same ones observed by Jeng [14] with the SMMT for most of the same reference beams. 

From the above, it can be stated that the modifications adopted in this study to extend the GSVATM for RC hollow beams under torsion, based on the proposed modifications for the SMMT by Jeng in 2015 [14], are valid. 

## 6. Conclusions

In this article the Generalized Softened Variable Angle Truss Model (GSVATM) was extended to cover RC hollow beams under torsion. The changes in the analytical model to consider the particular behavior of RC hollow beams for low loading levels, and based on the proposals to extend the Softened Membrane Model for Torsion (SMMT) [8,14], were presented. To assess the extended GSVATM, the theoretical predictions were compared with experimental results of several reference RC hollow beams under torsion.

From the obtained results, the following can be stated:

- It was found that the extended GSVATM captures well the full torsional response of RC hollow beams under torsion for all loading levels, although a drop of the torque right after the concrete cracking is predicted for most of the beams. This last observation is due to the shape of the smeared constitutive relationship used for the tensile concrete;
- Both the cracking and ultimate torque, which are important parameters for design, are very well predicted by the analytical model;
- The extended model still don´t predict very well the twists corresponding to the cracking and ultimate torque. However, since such parameters are not very important for the design, these results can be considered less important;

All structural members must be checked or designed for both the ultimate and service limit states. For this last one, verifications include the deformation of the beam, the cracking level and the stress/strain levels in the materials. Although general design rules of current codes of practice include orientations to assess structural beams for both the service (low) and ultimate (high) loads, specific design rules for torsion are still mainly focused on the ultimate limit state.

From the results of this study, it can be stated that the extended GSVATM is a reliable analytical model to predict the effective response of RC hollow beams under torsion, namely the *M_T_* − θ curve for all loading stages. In addition, the GSVATM can also provide reliable information for other parameters, such as the torsional stiffness and stress/strain in the materials for all loading stages, as well as information about the torsional ductility at the ultimate state. As a result, the GSVATM can be used as a reliable analytical tool by structural engineers to check and design more efficiently RC solid and hollow beams for both the torsional ultimate and service limit states.

## Figures and Tables

**Figure 7:**
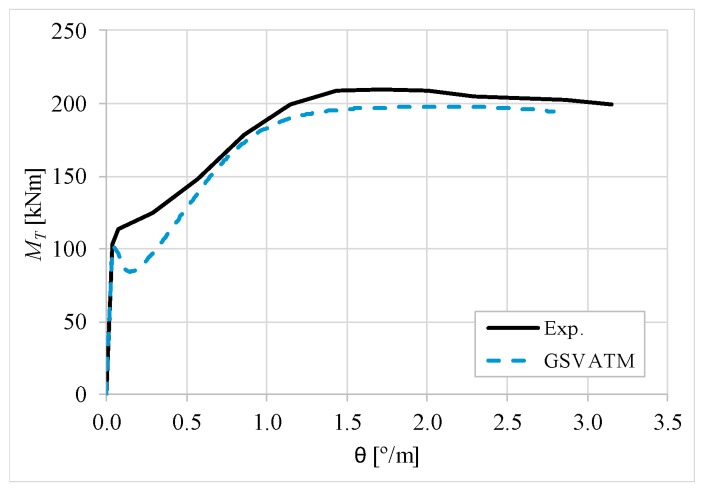
$M_{T}$−θ curves for Beam A095C.

**Figure 8:**
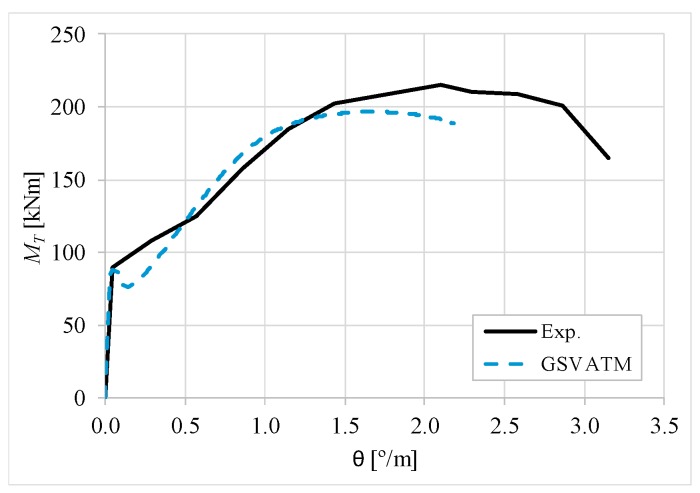
$M_{T}$−θ curves for Beam A120a.

**Figure 10:**
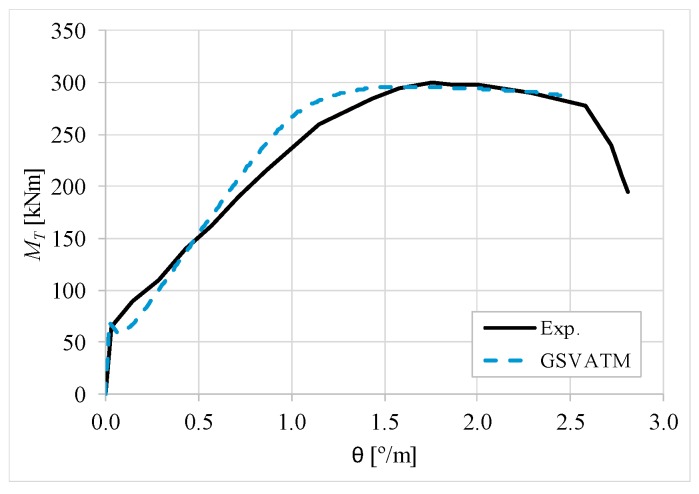
$M_{T}$−θ curves for Beam B080a.

**Figure 11:**
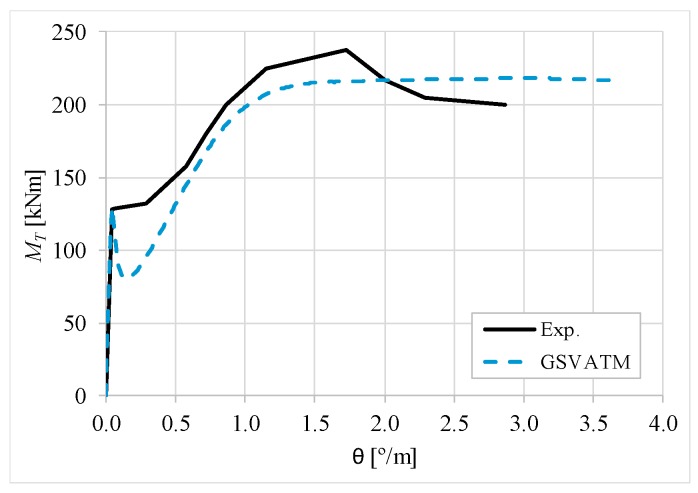
$M_{T}$−θ curves for Beam B110a.

**Figure 13:**
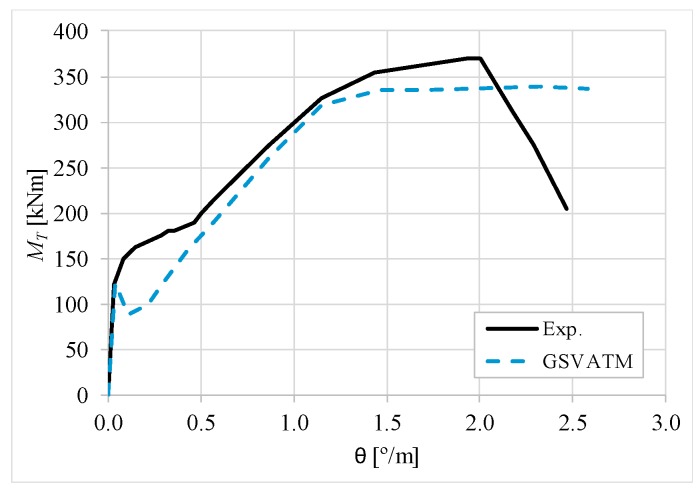
$M_{T}$−θ curves for Beam C100a.

**Figure 14:**
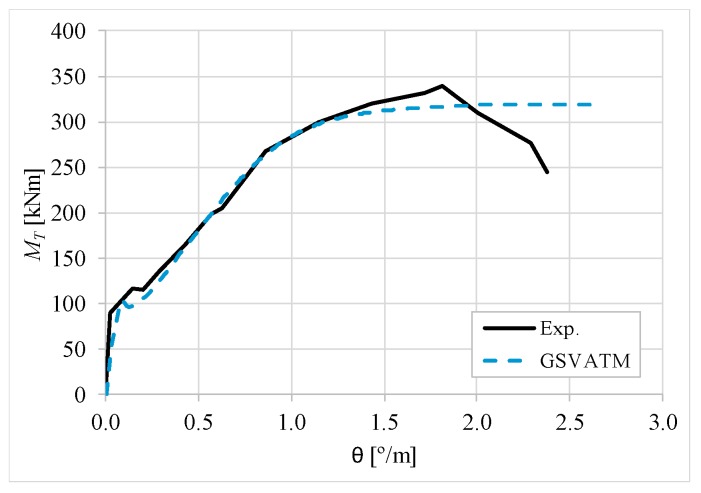
$M_{T}$−θ curves for Beam D075a.

**Figure 15:**
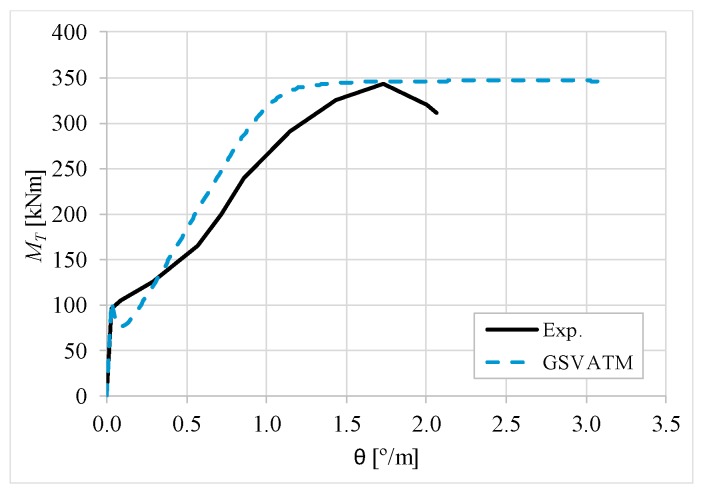
$M_{T}$−θ curves for Beam D090a.

**Figure 16:**
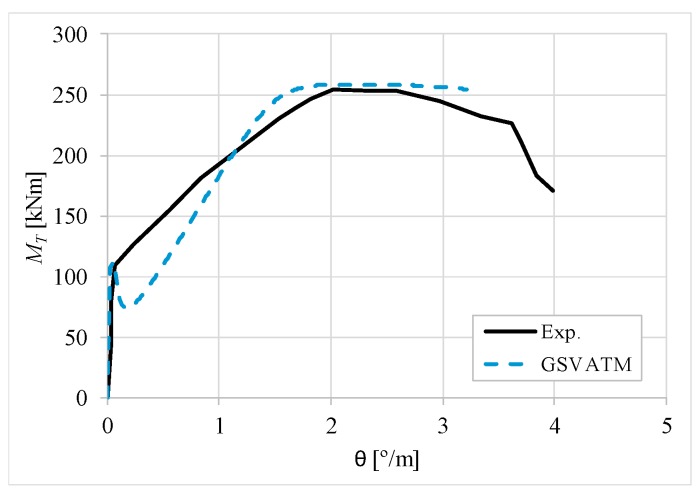
$M_{T}$−θ curves for Beam A2.

**Figure 17:**
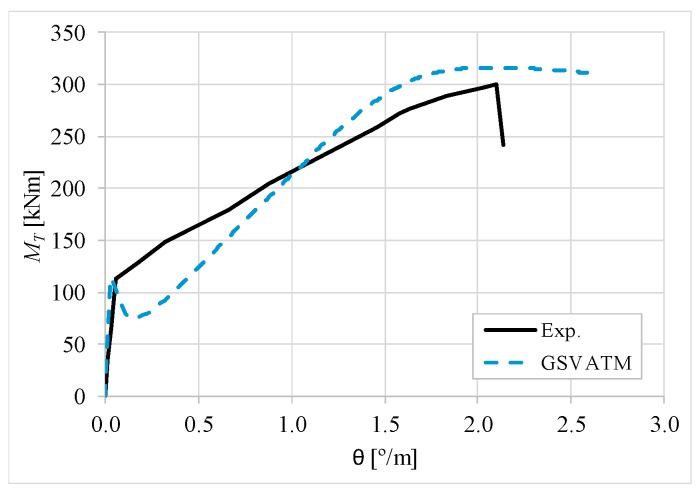
$M_{T}$−θ curves for Beam A3.

**Figure 18:**
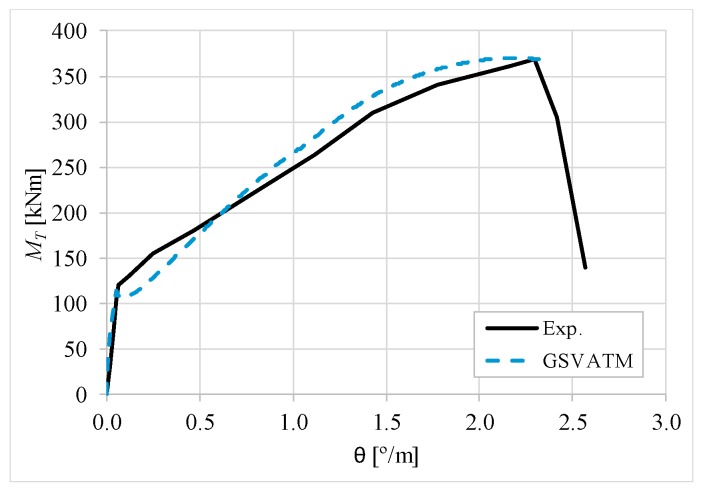
$M_{T}$−θ curves for Beam A4.

**Figure 19:**
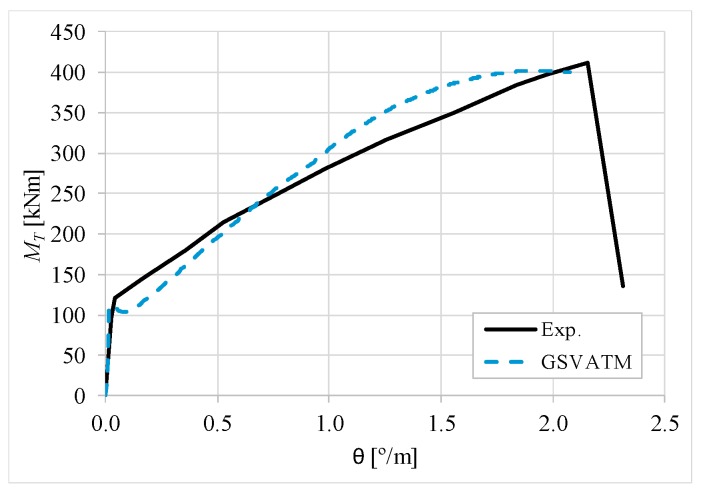
$M_{T}$−θ curves for Beam A5.

**Figure 20:**
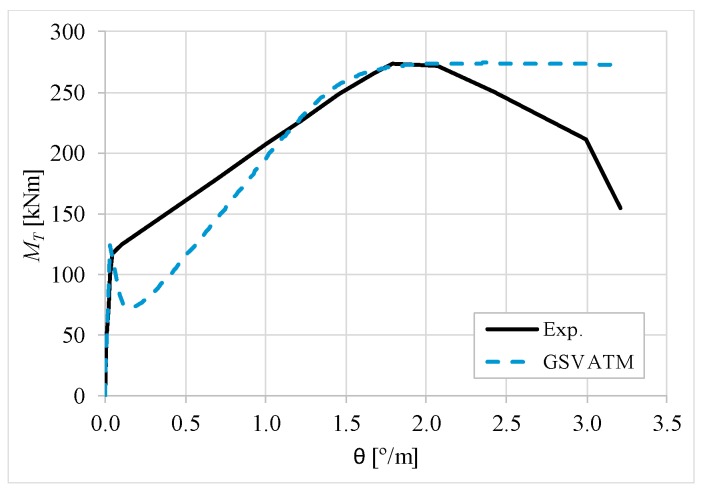
$M_{T}$−θ curves for Beam B2.

**Figure 22:**
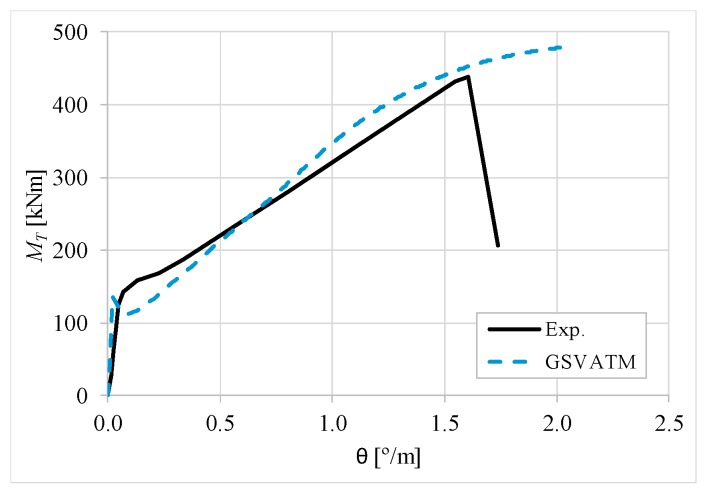
$M_{T}$−θ curves for Beam B4.

**Figure 23:**
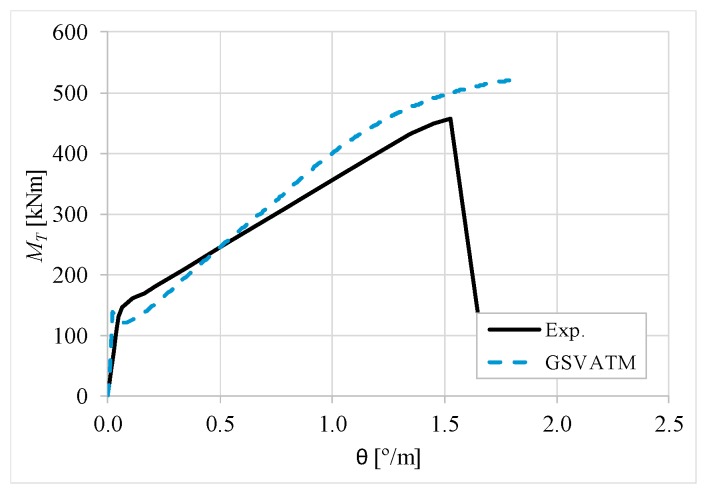
$M_{T}$−θ curves for Beam B5.

**Figure 24:**
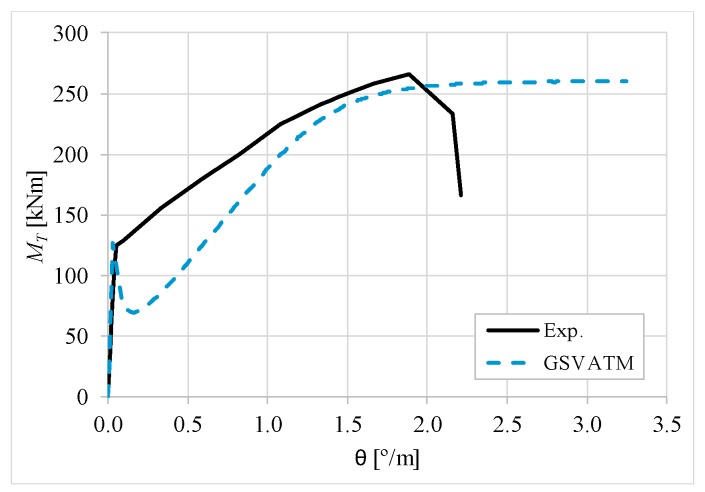
$M_{T}$−θ curves for Beam C2.

**Figure 26:**
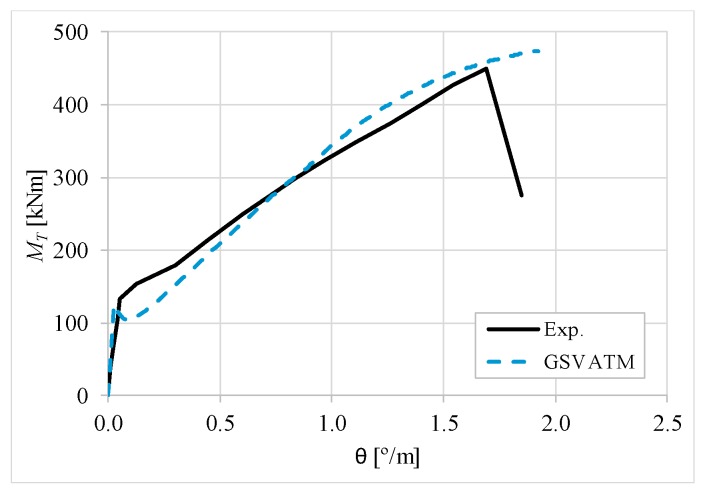
$M_{T}$−θ curves for Beam C4.

**Figure 27:**
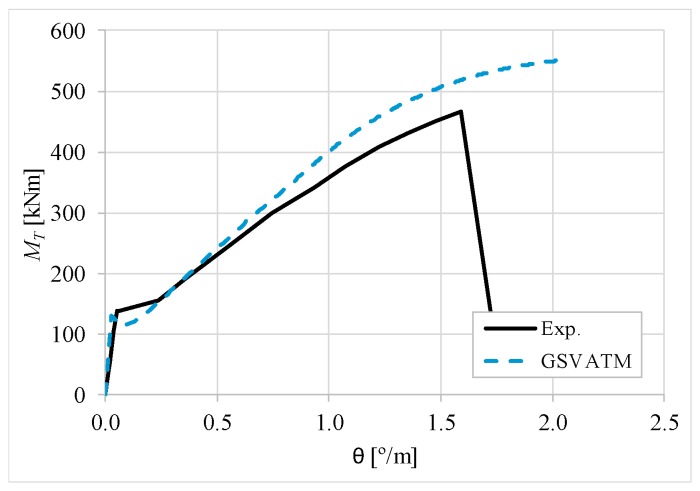
$M_{T}$−θ curves for Beam C5.

**Figure 28:**
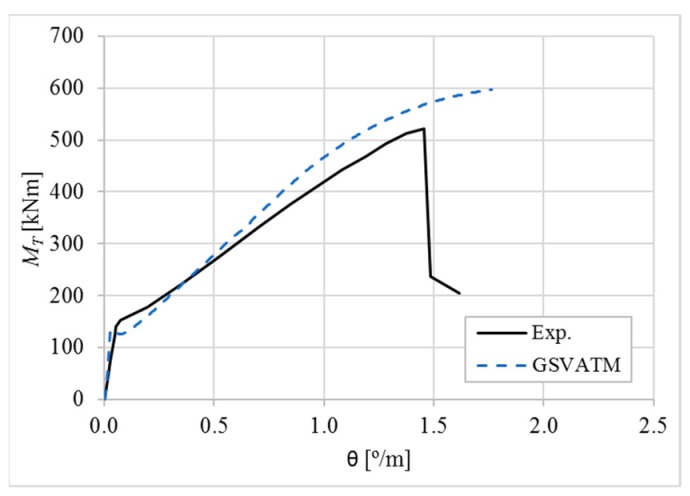
$M_{T}$−θ curves for Beam C6.

**Figure 29:**
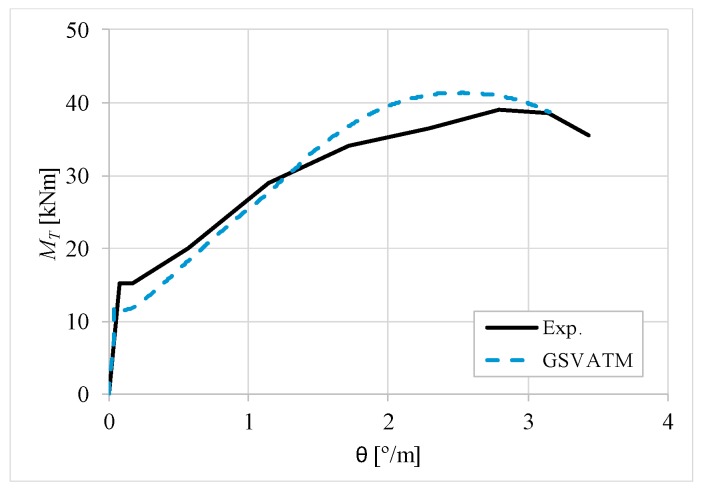
$M_{T}$−θ curves for Beam D3.

**Figure 30:**
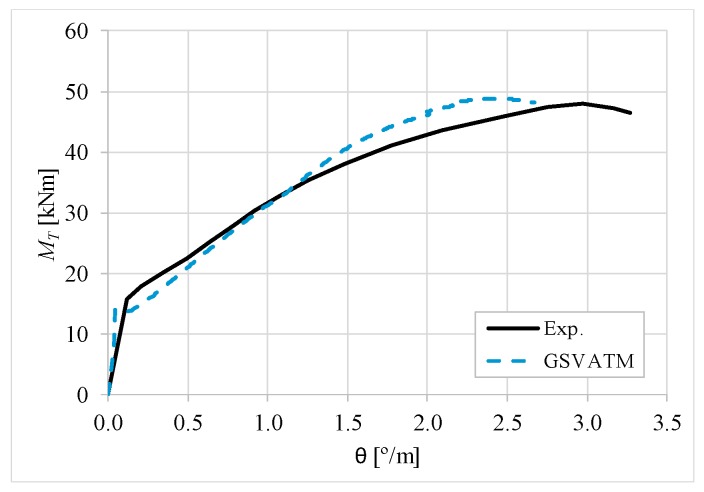
$M_{T}$−θ curves for Beam D4.

**Figure 31:**
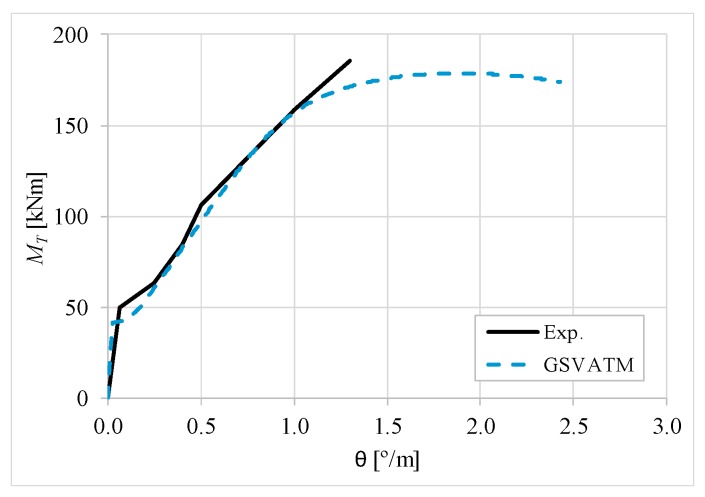
$M_{T}$−θ curves for Beam T0.

**Figure 32:**
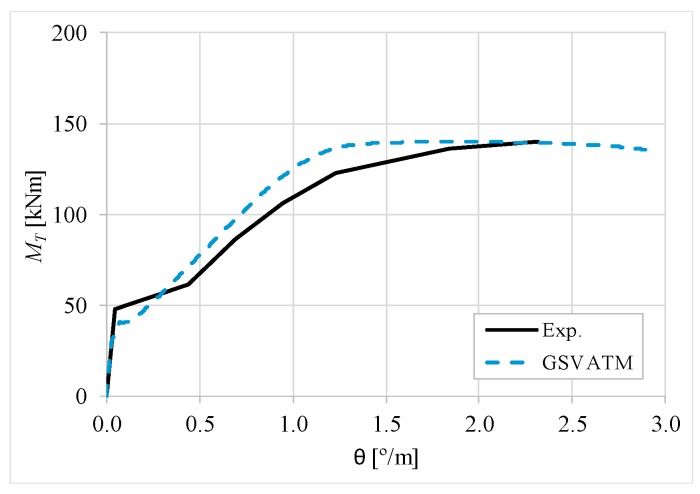
$M_{T}$−θ curves for Beam T1.

**Figure 33:**
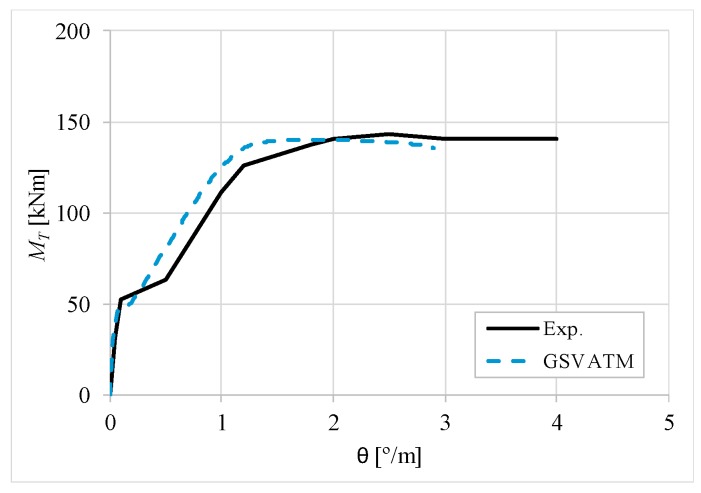
$M_{T}$−θ curves for Beam T2.

**Figure 34:**
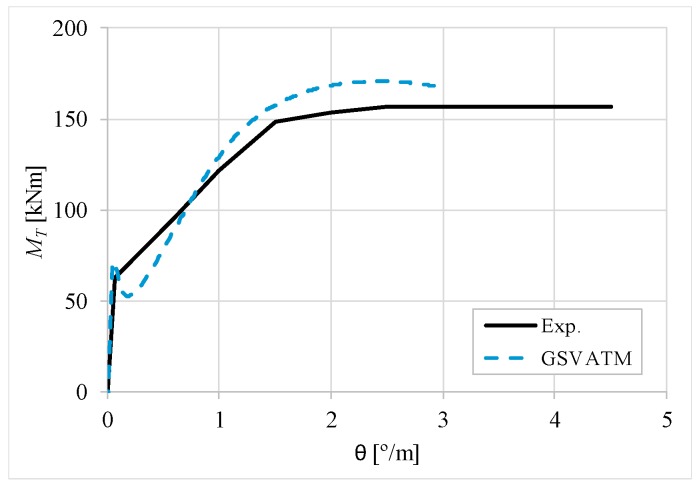
$M_{T}$−θ curves for Beam T5.

**Figure 35:**
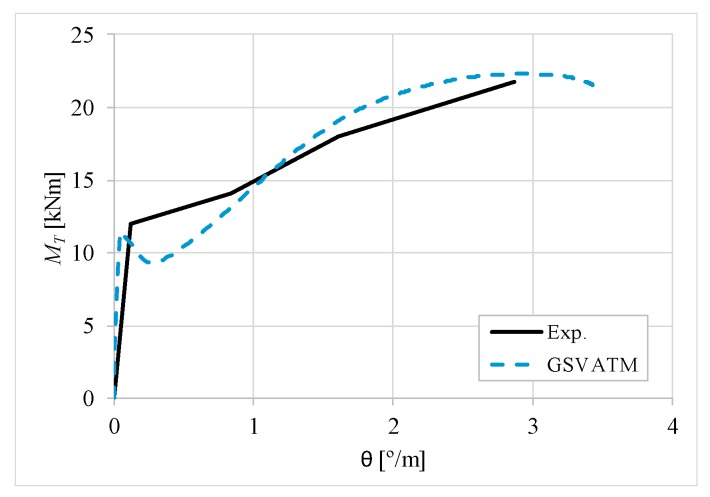
$M_{T}$−θ curves for Beam VH1.

**Figure 36:**
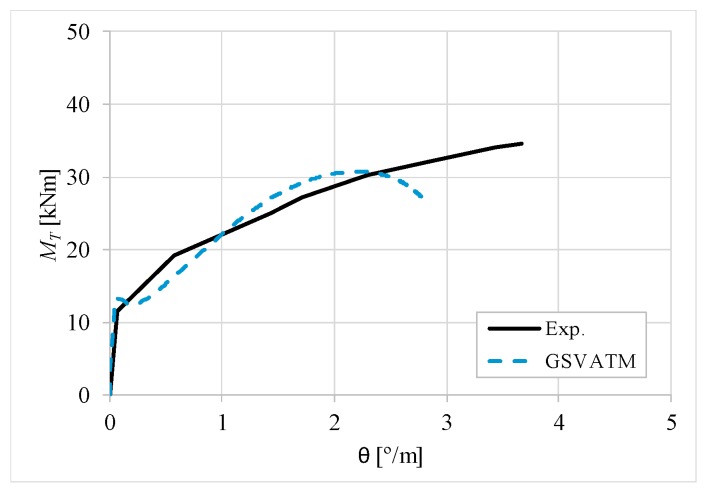
$M_{T}$−θ curves for Beam VH2.

**Table 1:** Properties of reference RC hollow beams.

Beam	*t* cm	*x* cm	*y* cm	*x*_1_ cm	*y*_1_ cm	*A_sl_* cm^2^	*A_st_*/*s* cm^2^/m	ρ*_l_* %	ρ*_t_* %	*f_ly_* MPa	*f_ty_* MPa	*f_c_* MPa
A095c [14]	14.5	49.7	71.1	43.7	65.1	13.16	9.93	0.372	0.611	371	381	35.1
A120a [14]	18.4	50.2	71.9	44.2	65.9	20.00	7.59	0.554	0.463	464	380	27.6
B065b [14]	9.2	50.3	71.0	44.3	65.0	50.97	9.93	1.427	0.608	452	380	39.2
B080a [14]	11.2	50.0	72.1	44.0	66.1	28.39	12.90	0.787	0.788	454	392	46.5
B110a [14]	15.5	49.8	71.0	43.8	65.0	20.00	8.60	0.566	0.529	453	369	48.1
C065a [14]	8.5	49.5	78.1	43.5	72.1	20.00	9.93	0.517	0.594	338	376	78.8
C100a [14]	12.7	49.9	72.3	43.9	66.3	28.39	12.90	0.787	0.788	466	447	90.6
D075a [14]	8.7	49.8	73.4	43.8	67.4	28.39	12.90	0.777	0.785	469	381	94.9
D090a [14]	10.5	50.1	72.2	44.1	66.2	28.39	12.90	0.785	0.787	466	447	105.7
A2 [22]	10.7	60.0	60.0	53.8	53.1	13.95	6.28	0.387	0.373	672	696	47.3
A3 [22]	10.9	60.0	60.0	54.0	53.5	18.10	8.27	0.503	0.494	672	715	46.2
A4 [22]	10.4	60.0	60.0	52.0	52.5	23.75	11.22	0.660	0.651	724	715	54.8
A5 [22]	10.4	60.0	60.0	52.8	52.8	30.66	14.14	0.852	0.829	724	672	53.1
B2 [22]	10.8	60.0	60.0	53.3	53.4	14.58	6.70	0.405	0.397	672	696	69.8
B3 [22]	10.9	60.0	60.0	53.5	53.7	23.75	11.22	0.660	0.668	724	715	77.8
B4 [22]	11.2	60.0	60.0	52.3	53.6	32.17	15.08	0.894	0.886	724	672	79.8
B5 [22]	11.7	60.0	60.0	51.8	51.8	40.21	18.85	1.117	1.085	724	672	76.4
C2 [22]	10.0	60.0	60.0	53.2	53.3	13.95	6.28	0.387	0.372	672	696	94.8
C3 [22]	10.3	60.0	60.0	54.5	54.0	23.75	10.47	0.660	0.631	724	715	91.6
C4 [22]	10.3	60.0	60.0	54.6	54.5	30.66	14.14	0.852	0.857	724	672	91.4
C5 [22]	10.4	60.0	60.0	54.0	54.3	36.69	17.40	1.019	1.047	724	672	96.7
C6 [22]	10.4	60.0	60.0	53.3	52.9	48.25	22.62	1.340	1.335	724	672	87.5
D3 [9]	6.4	25.4	38.1	21.6	34.3	11.36	10.16	1.173	1.173	341	333	28.4
D4 [9]	6.4	25.4	38.1	21.6	34.3	15.48	14.01	1.600	1.618	330	333	30.6
T0 [23]	8.0	50.0	50.0	43.0	43.0	32.16	10.28	1.286	0.707	345	357	45.1
T1 [23]	8.0	50.0	50.0	45.4	45.4	18.10	10.28	0.724	0.747	357	357	35.3
T2 [23]	8.0	50.0	50.0	43.0	43.0	18.10	10.28	0.724	0.707	357	357	35.3
T5 [23]	8.0	80.0	40.0	73.0	33.0	10.00	10.28	0.313	0.681	529	513	47.1
VH1 [24]	8.0	32.4	32.4	30.4	30.4	3.46	2.88	0.329	0.334	447	447	17.2
VH2 [24]	8.0	32.4	32.4	30.4	30.4	6.91	5.76	0.658	0.667	447	447	17.2

**Table 2:** Comparative analysis: cracking and ultimate points.

Beam	$M_{Tcr,\exp}\;\mathrm{kNm}$	$M_{Tcr,th}\;\mathrm{kNm}$	$\frac{M_{Tcr,exp}}{M_{Tcr,th}}$	$\theta_{cr,\exp}{}^{\circ}/m$	$\theta_{cr,th}{}^{\circ}/m$	$\frac{\theta_{cr,\exp}}{\theta_{cr,th}}$	$M_{Tu,\exp}\;\mathrm{kNm}$	$M_{Tu,th}\;\mathrm{kNm}$	$\frac{M_{Tu,\exp}}{M_{Tu,th}}$	$\theta_{u,\exp}\text{°}/m$	$\theta_{u,th}\;/m$	$\frac{\theta_{u,\exp}}{\theta_{u,th}}$
A095c [14]	102.88	101.6	1.013	0.034	0.042	0.810	209.98	197.49	1.063	1.714	2.205	0.777
A120a [14]	89.78	88.38	1.016	0.048	0.042	1.143	215.25	196.61	1.095	2.1	1.674	1.254
B065b [14]	54.43	52.66	1.034	0.034	0.02	1.700	265	242.84	1.091	1.203	1.397	0.861
B080a [14]	65.24	63.97	1.020	0.033	0.045	0.733	300.66	295.9	1.016	1.756	1.611	1.090
B110a [14]	128.3	129.52	0.991	0.041	0.045	0.911	237.48	218.06	1.089	1.723	3.016	0.571
C065a [14]	91.68	96.32	0.952	0.029	0.033	0.879	-	217.81	-	-	3.527	-
C100a [14]	122.23	122.71	0.996	0.029	0.036	0.806	370.15	338.76	1.093	1.927	2.323	0.830
D075a [14]	90.09	103.95	0.867	0.028	0.082	0.341	339.48	319.11	1.064	1.815	2.364	0.768
D090a [14]	96.05	103.56	0.927	0.029	0.027	1.074	343.08	346.61	0.990	1.734	2.597	0.668
A2 [22]	109.49	112.02	0.977	0.064	0.053	1.208	254.08	258.7	0.982	2.019	2.178	0.927
A3 [22]	113.27	114.58	0.989	0.057	0.031	1.839	299.91	316.37	0.948	2.101	2.075	1.013
A4 [22]	120.87	114.29	1.058	0.063	0.048	1.313	368.22	369.96	0.995	2.295	2.196	1.045
A5 [22]	120.93	110.65	1.093	0.044	0.018	2.444	412.24	402.28	1.025	2.154	1.939	1.111
B2 [22]	116.72	124	0.941	0.044	0.032	1.375	273.27	274.07	0.997	1.787	2.39	0.748
B3 [22]	130.45	127.5	1.023	0.045	0.032	1.406	-	426.63	-	-	2.195	-
B4 [22]	142.93	136.19	1.049	0.07	0.025	2.800	437.85	478.55	0.915	1.605	2.03	0.791
B5 [22]	146.26	138.22	1.058	0.064	0.023	2.783	456.19	522.07	0.874	1.526	1.837	0.831
C2 [22]	124.46	126.53	0.984	0.049	0.033	1.485	266.14	259.94	1.024	1.884	3.143	0.599
C3 [22]	131.93	129.74	1.017	0.064	0.033	1.939	-	408.54	-	-	2.236	-
C4 [22]	124.77	102.12	1.222	0.051	0.026	1.962	450.3	474.3	0.949	1.693	1.922	0.881
C5 [22]	138.34	131.07	1.055	0.051	0.026	1.962	467.26	550.51	0.849	1.59	2.026	0.785
C6 [22]	139.09	134.39	1.035	0.054	0.026	2.077	521.33	597.04	0.873	1.456	1.765	0.825
D3 [9]	15.15	12.01	1.261	0.081	0.039	2.077	39.11	41.34	0.946	2.795	2.526	1.106
D4 [9]	15.82	14.08	1.124	0.118	0.042	2.810	47.92	48.77	0.983	2.97	2.453	1.211
T0 [23]	49.82	41.86	1.190	0.062	0.025	2.480	185.5	171.83	1.080	1.3	1.841	0.706
T1 [23]	47.99	41.15	1.166	0.043	0.071	0.606	140.01	140.04	1.000	2.316	1.835	1.262
T2 [23]	52.79	46.15	1.144	0.098	0.071	1.380	143.1	140.18	1.021	2.5	1.743	1.434
T5 [23]	62.54	71.98	0.869	0.063	0.049	1.286	156.88	170.68	0.919	2.5	2.436	1.026
VH1 [24]	11.99	11.41	1.051	0.123	0.044	2.795	21.79	20.3	1.073	2.865	2.967	0.966
VH2 [24]	11.5	13.26	0.867	0.072	0.045	1.600	34.5	30.73	1.123	3.667	2.186	1.677
		$\bar{x}=$	1.033		$\bar{x}=$	1.601		$\bar{x}=$	1.003		$\bar{x}=$	0.954
		s=	0.098		s=	0.711		s=	0.075		s=	0.258
		cv=	9.46%		cv=	44.42%		cv=	7.52%		cv=	27.03%
